# Synthesis of Ring II/III Fragment of Kanamycin: A New Minimum Structural Motif for Aminoglycoside Recognition

**DOI:** 10.3390/antibiotics8030109

**Published:** 2019-08-02

**Authors:** Sandra G. Zárate, Agatha Bastida, Andrés G. Santana, Julia Revuelta

**Affiliations:** 1 Instituto de Química Orgánica General, CSIC, Juan de la Cierva 3, 28006 Madrid, Spain; 2 Facultad de Tecnología, Carrera de Ingeniería Química, Universidad Mayor, Real y Pontificia de San Francisco Xavier de Chuquisaca, Regimiento Campos 180, Casilla 60-B Sucre, Bolivia

**Keywords:** aminoglycoside antibiotics, enzymatic resistance, minimum structural motif, oligosaccharides, pseudo-disaccharide

## Abstract

A novel protocol has been established to prepare the kanamycin ring II/III fragment, which has been validated as a minimum structural motif for the development of new aminoglycosides on the basis of its bactericidal activity even against resistant strains. Furthermore, its ability to act as a AAC-(6′) and APH-(3′) binder, and as a poor substrate for the ravenous ANT-(4′), makes it an excellent candidate for the design of inhibitors of these aminoglycoside modifying enzymes.

## 1. Introduction

Since the discovery of streptomycin in 1944 [1], aminoglycosides have found widespread clinical use due to its broad antimicrobial spectrum and rapid bactericidal effects [2,3]. Their mode of action involves binding to the 16S ribosomal subunit at the t-RNA acceptor A-site (aminoacyl site), where they interfere with the ability of the ribosome to correctly decode mRNA during protein synthesis [4,5,6,7]. Unfortunately, irreversible ototoxic side effects and growing bacterial resistance have narrowed the significance of aminoglycosides as antibiotics in the clinical practice [8].

**Figure 1:**
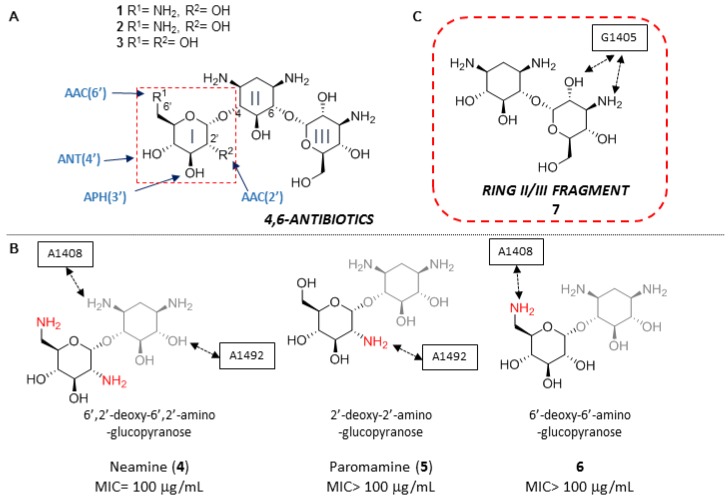
(**A**) Structure of kanamycin A (**1**), kanamycin B (**2**) and kanamycin C (**3**). (**B**) Ring I/II fragments (**4**–**6**) of kanamycins. The functional relevance of the drug/RNA contacts established through ring I amines are indicated in each case. Besides, typical ranges for the MIC values of natural kanamycins (**1**–**3**) and of the fragments thereof (**4**–**6**) are also presented. (**C**) Ring II/III fragment studied in this paper (**7**).

Kanamycins (1–3) are natural antibiotics belonging to a group of aminoglycosides containing a 4,6-disubstituted 2-deoxystreptamine (ring II) core and have been used against both Gram-(+) and Gram-(−) bacteria for more than fifty-years (Figure 1A) [9]. The pseudo-disaccharide I/II fragments (4–6) of this family of antibiotics, with slightly different OH/NH_2_ patterns in unit I, is common to most aminoglycosides and have been considered until now essential for specific complex formation, and consequently, for antibiotic activity (Figure 1B) [10]. For this reason, most approximations to new aminoglycosides described to date involve the synthesis of derivatives maintaining the I/II core and eliminating or modifying ring III [11,12,13].

Unfortunately, there are many aminoglycoside-modifying enzymes transferring acetyl, phosphoryl and adenyl groups in a cofactor-dependent manner to virtually every amino or hydroxyl substituent of the I/II structural motif (Figure 1A) [14]. As a result, semisynthetic congeners of the 4,6-disubstituted 2-deoxystreptamine class were developed to overcome the inactivating action of a subset of the aforementioned enzymes and their different isoforms prevalent in other pathogens [15,16,17,18]. Despite the urgent need of new anti-infective therapies, it should be noted that no new aminoglycoside antibiotic was introduced since the early 1980s [19], up until this year, when promising semisynthetic propylamycin was reported by Crich and coworkers [20].

A general way for the discovery of new aminoglycosides can be thus summarized on keeping a minimum structural motif (MSM) that provides basic affinity and, subsequently, hit-to-lead optimization of the resulting structure. Herein, we report on the synthesis of novel pseudo-disaccharide 7 that shows interesting properties as a novel MSM, while still retaining a considerable part of the antibiotic activity against several strains, including some expressing the most clinically relevant aminoglycoside modifying enzymes (Figure 1A).

## 2. Results and Discussion

### 2.1. Synthesis of Pseudo-Disaccharide ***7***

**Scheme 1:**
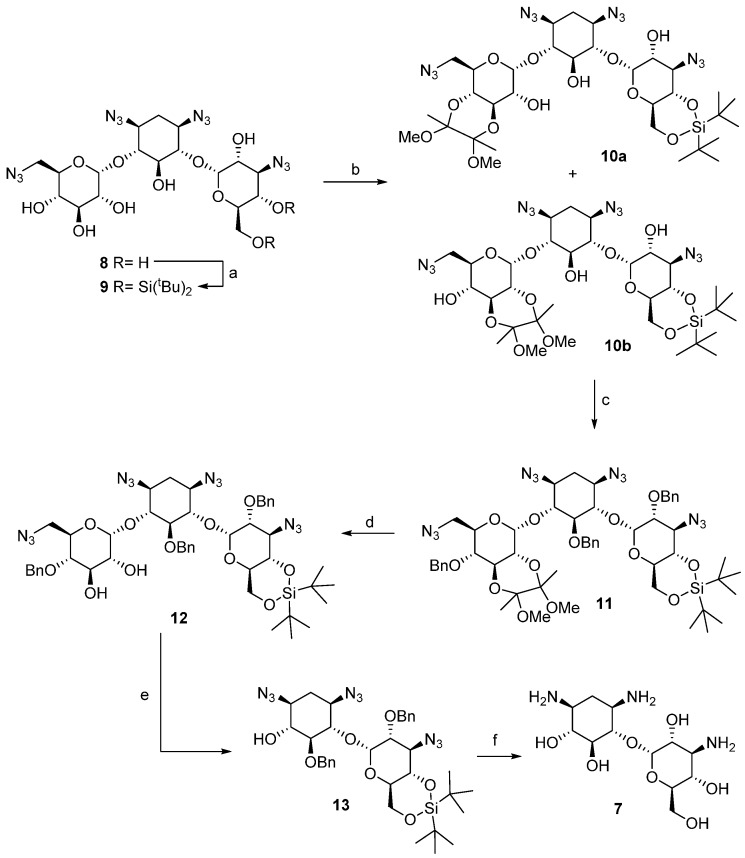
Reagents and conditions: (a) tBu_2_Si(OTf)_2_, DMAP, py, r.t., 24 h., 80%; (b) 2,2,3,3-tetramethoxybutane, CH(OCH_3_)_3_, 2,3-butanodione, p-TosOH, MeOH, reflux, 12 h., 77%; (c) BnBr, TBAI, NaH, DMF, r.t., 24 h., 65%; (d) TFA:H_2_O (1:1), r.t.; 40 min.; 75%; (e) i. NaIO_4_, MeOH, 0 °C, 12 h., ii. TEA, THF, r.t., 12 h., 88% (two steps); (f) i. TBAF, THF, r.t., 24 h., ii. PMe_3_, NaOH (0.1 M), THF, 55 °C, 12 h., iii. H_2_, Pd(OH)_2_·C, H_2_O/TFA (1:1), r.t., 12 h. 47% (three steps).

The preparation of pseudo-disaccharide **7** was carried out according to the sequence described in Scheme 1. Our synthetic effort began with the preparation of tetraazidokanamycin A (**8**), an intermediate suitably protected with azide groups, according with the procedure described previously by our laboratory [21]. As a next step, simultaneous regioselective protection of C-6″ and C-4″ hydroxyl groups was achieved by treatment of **8** with di-*tert*-butylsilyl bis(trifluoromethanesulfonate) in pyridine to afford compound **9** in 80% yield [22]. The protection of C-6″ OH was proven to be necessary for minimizing the formation of byproducts and for allowing an easier purification after the regioselective protection of ring I, the key step of our strategy. After undergoing many frustrations attempting the regioselective protection of positions 2′ and 3′ in ring I of kanamycin A [23], we envisioned the use of 2,3-butanodione-bis-dimethyl acetal as a plausible strategy [24]. Indeed, this reaction afforded an equimolar mixture of **10a** and **10b**, by protection of C-3′ and C-4′ hydroxyl groups and C-2′ and C-3′, respectively, decreasing the total yield of the synthesis considerably given that **10a** does not undergo the required β-elimination after periodate oxidation of the remaining diol. Despite this setback, this is to date the best procedure found for the selective protection of 2’ and 3’ hydroxyl groups of kanamycin A (**1**). In a next step, Williamson benzylation of the rest of the hydroxyl groups in compound **10b** afforded **11** in 65% yield. Finally, the regioselective deprotection of the *O*-2′,3′-butanodione-bis-dimethyl acetal using TFA provided diol **12** in 85% yield, the key intermediate in the preparation of **7**.

Oxidation of **12** was found to proceed efficiently with a moderate excess of sodium periodate in THF [25]. Treatment of the resulting dialdehyde intermediate in methanol solution with triethylamine effected the desired β-elimination to give a solid product that was assigned as the expected pseudo-disaccharide structure **13**. Finally, cleavage of the silyl-acetal function in the presence of TBAF, removal of the azide groups by Staudinger reduction and subsequent catalytic hydrogenolysis gave the desired pseudo-disaccharide **7** (Supplementary Material, Figure S1).

### 2.2. Antibiotic Activity and Resistance Enzyme Susceptibility of ***7***

To assess the effect that ring I cleavage exhibits on the antibacterial capacity, minimum inhibitory concentration (MIC) values of the parent kanamycin A (**1**) and the fragments neamine (**4**) and pseudo-disaccharide **7**, were measured against a battery of antibiotic non-resistant and resistant strains (Table 1).

According to the obtained data, pseudo-disaccharide **7** showed an expected drop in efficacy (MIC = 50–100 μg mL^−1^) in comparison with the natural kanamycin A **1** (MIC = 1.5–6 μg mL^−1^). Remarkably, derivative **7** still represents an improvement over neamine (**4**), lowering the MIC value from 100 μg mL^−1^ to 50 μg mL^−1^. Interestingly, according to the MIC values measured for compound **7** (Table 1, entries 5–6), while a complete loss of activity for antibiotic **1** and for neamine (**4**) was observed, fragment **7** maintains some activity against aminoglycoside inactivation performed by APH-(3′) and AAC-(6′). These results are in agreement with the relative enzymatic activity observed: for APH-(3′) and for AAC-(6′) the rate of phosphorylation/acetylation (V_rel_(**7**)/V_rel_(**1**)) is zero, indicating that compound **7** is not inactivated by these enzymes. However, in the case of ANT(4′), adenylation was much less effective [(V_rel_(**5**)/V_rel_(**1**) = 0.11], whereby position 4″ of original III ring of **1** is being slowly modified [26]. This is in agreement with results previously described by our group, where we demonstrated that ANT-(4′) exhibits a remarkably low sensitivity toward the drug global shape and represents a paradigmatic example of substrate promiscuity [27].

Finally, we evaluated the capacity of kanamycin A (**1**), neamine (**4**) and pseudo-disaccharide **7** to bind the aforementioned enzymes ANT-(4′), APH-(3′) and AAC-(6′) employing thermal melting shift experiments (Table 2). The change in unfolding transitions temperature (ΔTm) in the presence and in the absence of the ligands provides an estimation of the ligand/protein complex stability. Surprisingly, compound **7** proved to be an appropriate ligand not only for ANT-(4′), but also for AAC-(6′) and APH-(3′) even through it is not a substrate of these latter enzymes, producing clear thermal stabilizations of all of them (ΔTm = 5–7 °C).

In conclusion, and considering the kinetic parameters, the estimated ligands binding affinity and the MIC values, compound **7** maintains a bactericidal activity even in resistant strains unlike neamine (**4**), where AAC-(6′) and APH-(3′) were shown unable to modify the substrate and ANT-(4′) only barely, while in all three cases **7** is a ligand of the enzymes. These results strongly suggest that compound **7** should be carefully considered in the design of novel antibiotics with improved activity against resistant strains and inhibitors of these aminoglycoside modifying enzymes.

## 3. Materials and Methods

### 3.1. General Procedures

All reactions were carried out in oven-dried glassware under a positive pressure of argon unless otherwise noted. Neomycin B and Kanamycin A free bases were prepared from the corresponding monosulfate salts (purchased from Santa Cruz Biotechnology, inc. and Sigma-Aldrich, respectively) by use of Amberlite-IRA 400 (OH^−^) strongly basic ion-exchange resin. Solvents were dried in a Pure Solv system model PS-400-3-MD. Reactions were monitored by analytical thin-layer chromatography (TLC) on EM silica gel 60 F254 plates (0.25 mm), visualized by ultraviolet light and/or by staining with ceric ammonium molybdate, H_2_SO_4_ or ninhydrin. Column chromatography was performed on Silice 60 (230–400 µM) and on Amberlite CG-50 (NH4^+^) cation exchange resin. ^1^H NMR spectra were recorded on a Varian Inova-400 (400 MHz) and Varian UNITY 500 (500 MHz) in CDCl_3_, CD_3_OD and D_2_O solutions at ambient temperature. Data were reported as follows: chemical shift on the δ scale (either using TMS or residual proton solvent as internal standard), multiplicity (br = broad, s = singlet, d = doublet, t = triplet, q = quartet, m = multiplet), coupling constant(s) in hertz, and integration. ^13^C NMR spectra were recorded on a Varian Inova-400 (100 MHz) and Varian UNITY 500 (125 MHz). Mass spectra were recorded on an AGILENT 6520 Accurate-Mass QTOF LC/MS spectrometer using the electrospray modes (ES).

### 3.2. Synthesis of 4-O-(2,6-di-Deoxy-α-d-2,6-di-amine-glucopyranosyl)-2-deoxy-streptamine (Neamine) (***4***)

Neomycin B free base (5.0 g, 5.5 mmol) was suspended in a mixture of H_2_O/MeOH (80 mL, 1:1 *v*/*v*) and concentrated HCl (16 mL) was added. The mixture was stirred overnight at 75 °C and then the solvent was eliminated under reduced pressure. The obtained residue was re-suspended again in a mixture of H_2_O/MeOH (80 mL, 1:1 *v*/*v*) and the reaction was heated at 75 °C for 12 h. Finally, the solvent was partially eliminated under vacuum (until approximately 20 mL), being observed the formation of a precipitate of **4**, which was finally filtrated and washed with cold Et_2_O to give pure neamine **4** hydrochloride (2.3 g, 89%) as a white solid. ^1^H NMR (D_2_O, 400 MHz) δ: 5.90 (d, *J* = 3.7 Hz, 1H), 4.03–3.92 (m, 3H), 3.68 (t, *J* = 9.9 Hz, 1H), 3.59–3.24 (m, 7H), 2.49 (dt, *J* = 12.6, 4.3 Hz, 1H), 1.89 (q, *J* = 12.6 Hz, 1H). ^13^C NMR (D_2_O, 100 MHz) δ: 98.1, 79.7, 77.2, 74.5, 72.7, 71.2, 70.3, 55.5, 51.7, 50.5, 42.1, 30.3. MS-API-ES (**4·**HCl): 323 [M + H]^+^. HRMS (ESI+) calc for C_12_H_27_N_4_O_6_ 323.19251, found 323.19199.

### 3.3. Synthesis of 6-O-[(3-Deoxy-3-amino)-α-d-glucopyranosyl]-2-deoxy-streptamine (***7***)

#### 3.3.1. Synthesis of 1,3,6′,3″-Tetra-azido-kanamycin A (**8**)

Kanamycin A free base (**1**) (1.35 g, 2.8 mmol) and the catalyst CuSO_4_·5H_2_O (7 mg, 0.028 mmol) were dissolved in water (8.2 mL) and sodium hydrogencarbonate (0.94 g, 11.2 mmol) was added. Finally, fresh triflyl azide solution (14 mL of a 2M solution in toluene, 28 mmol) [28] was added at once with vigorous stirring, followed by a dropwise addition of a mixture of MeOH: pyridine (1/1 *v*/*v*, 49.2 mL). The mixture was stirred until TLC (CH_2_Cl_2_: MeOH, 9:1, R*_f_* = 0.3) showed the reaction to be complete (18 h.). The solvent was removed under reduced pressure. Subsequently, the residue was treated with Ac_2_O/Py (1/2, *v*/*v*, 30 mL) and a catalytic amount of DMAP (17 mg, 0.14 mmol). The mixture was stirred for 12 h, the solvent was eliminated under vacuum and the residue was extracted with AcOEt (3 × 25 mL). The combined organic layers were dried over anhydrous Na_2_SO_4_, concentrated and the residue was purified by column chromatography on silica gel (hexane/ethyl acetate, 1/1) to give 2′,3′,4′,2″,4″,6″-*O*-hexaacetyl-1,3,6′,3″-tetraazido-kanamycin A (1.45 g, 60%) as a colorless foam. ^1^H NMR (500 MHz, CDCl_3_) δ 5.47 (m, 2H), 5.33 (d, *J* = 3.7 Hz, 1H), 5.05 (dd, *J* = 9.9 Hz, 1H), 4.95–4.89 (m, 2H), 4.78 (dd, 1H, *J* = 10.5, 3.7 Hz), 4.33 (ddd, *J* = 9.4, 5.3, 2.6 Hz, 1H), 4.28 (ddd, *J* = 9.9, 3.7 Hz, 1H), 4.13–4.06 (m, 2H), 4.09 (d, *J* = 10.5 Hz, 1H), 3.57 (ddd, *J* = 9.4, 3.1 Hz, 1H), 3.50–3.44 (m, 2H), 3.42–3.29 (m, 5H), 2.28 (ddd, *J* = 13.1, 4.4 Hz, 1H), 2.18 (s, 3H), 2.13 (s, 3H), 2.08 (s, 3H), 2.05 (s, 6H), 2.02 (s, 3H), 1.57 (q, *J* = 12.7 Hz, 1H).; ^13^C NMR (100 MHz, CDCl_3_) δ 170.3, 170.1, 170.0, 169.9, 169.8, 169.7, 98.0, 97.3, 85.6, 82.1, 74.0, 71.9, 71.1, 69.9, 69.2, 68.3, 68.1, 67.6, 63.0, 61.2, 60.5, 58.7, 51.5, 32.6, 20.8, 20.7, 20.6, 20.4, 20.2, 20.1. MS-API-ES: 863 [M + Na]^+^. Finally, this intermediate was de-O-acetylated by treating a solution of it (1.45 g, 1.72 mmol) with a 1M solution of MeONa in MeOH (17.2 mL, 17.2 mmol). The mixture was stirred under an Ar atomosphere for 8 h. The reaction mixture was neutralized with Amberlite^®^ IRA-120 (H^+^) to pH = 5, filtered and the resin was washed with MeOH (20 mL). The combined filtrates were concentrated and the residue was purified by flash chromatography on silica gel using CH_2_Cl_2_/MeOH, (9:1) to give **8** (0.91 g, 90%) as a white solid. ^1^H NMR (MeOD-d_4_, 400 MHz) δ: 5.24 (d, *J* = 3.8 Hz, 1H), 5.18 (d, *J* = 3.8 Hz, 1H), 4.08-3.98 (m, 2H), 3.78-3.28 (m, 15H), 2.33 (dt, *J* = 4.2, 12.6 Hz, 1H), 1.56 (q, *J* = 12.4 Hz, 1H). ^13^C NMR (MeOD-d_4_, 100 MHz) δ: 100.4, 98.0, 83.0, 80.2, 73.8, 72.9, 71.9, 71.5, 70.4, 70.1, 67.9, 66.4, 60.0, 58.8, 50.8, 31.5. MS-API-ES: 589 [M + H]^+^. HRMS (ESI+) calc for C_18_H_29_N_12_O_11_ 589.20733, found 589.20699.

#### 3.3.2. Synthesis of 4″,6″-*O*-di-*tert*-Butyl-silane-1,3,6′,3″-tetra-azido-kanamycin A (**9**)

To a well-stirred solution of **8** (0.91 g, 1.55 mmol) and DMAP (0.094 g, 0.77 mmol) in pyridine (3 mL) at −10 °C was added dropwise di-*tert*-butylsilyl bis(trifluoromethanesulfonate) (0.95 g, 2.17 mmol). The mixture was stirred at this temperature for 10 min and overnight at room temperature. Then MeOH (1 mL) was added and the mixture was concentrated. Next, a mixture of AcOEt (100 mL) and brine (100 mL) was added, the phases were separated and the aqueous phase was further extracted twice with AcOEt (100 mL). The combined organic phases were dried over anhydrous Na_2_SO_4_ and finally the solvent was removed under reduced pressure. Finally, the residue was purified by flash chromatography on silica gel using CH_2_Cl_2_/MeOH (9:1) to give **9** (0.9 g, 80%) as a white solid. ^1^H NMR (MeOD-d_4_, 300 MHz) δ 5,21 (d, *J* = 3.5 Hz, 2H), 4.53 (ddd, *J* = 5.1, 9.6, 9.6 Hz, 1H,), 4.16–4.00 (m, 2H), 3.83–3.30 (m, 14H), 2.40 (ddd, *J* = 4.0, 4.0, 8.5 Hz, 1H), 1.59 (q, *J* = 12.3, 1H), 1.20 (t, *J* = 7.0 Hz, 1H), 1.08 (s, 9H, Si), 1.03 (s, 9H, Si). ^13^C NMR (MeOD-d_4_, 75 MHz) δ: 102.9, 99.5, 86.1, 81.0, 78.4, 75.4, 74.8, 73.8, 73.4, 72.2, 71.6, 67.8, 67.6, 62.1, 60.6, 52.9, 33.3, 27.9, 27.8, 27.5, 23.4, 20.9. MS-API-ES: 751.3 [M + Na]^+^. HRMS (ESI+) calc for C_26_H_44_N_12_NaO_11_Si 751.2914, found 751.29108.

#### 3.3.3. Synthesis of 2′,3′-*O*-(2,3-Butanedione-bis-dimethyl-acetal)-4″,6″-*O*-di-*tert*-butyl-silane-1,3,6′,3″-tetra-azido-kanamycin A (**10a**) and 3′,4′-*O*-(2,3-Butanedione-bis-dimethyl-acetal)-4″,6″-*O*-di-*tert*-butyl-silane-1,3,6′,3″-tetra-azido-kanamycin A (**10b**)

To a well stirred solution of **9** (0.90 g, 1.24 mmol) in MeOH was added 2,2,3,3-tetra-methoxy-butane (0.44 g, 2.5 mmol), trimethyl orthoformate (0.54 mL, 5.0 mmol), 2,3-butanedione (0.12 mL, 1.37 mmol) and a catalytic amount of *p*-TosOH·H_2_O (31 mg, 0.18 mmol). The mixture was refluxed for 18 h under Ar. Subsequently, after the system reaches room temperature, solid NaHCO_3_ was added (~0.3 g) and the solvent was eliminated under reduced pressure. Then, a mixture of AcOEt (100 mL) and brine (100 mL) was added, the phases were separated and the aqueous phase was further extracted twice with AcOEt (100 mL). The combined organic phases were dried over anhydrous Na_2_SO_4_ and finally the solvent was eliminated under reduced pressure. The resulting residue was purified by flash chromatography on silica gel using hexane/AcOEt (6:4) to give **10a** (0.4 g, 39%) as a white foam and **10b** (0.4 g, 39%) as a white solid. **10a**: ^1^H NMR (CDCl_3_, 400 MHz) δ: 5.18 (d, *J* = 3.7 Hz, 1H), 5.09 (d, *J* = 3.8 Hz, 1H), 4.75 (sa, 1H, H-OH), 4.26 (ddd, *J* = 10.0, 10.0, 5.0 Hz, 1H), 4.17 (ddd, *J* = 2.5, 3.9, 10.0 Hz, 1H), 4.07(dd, *J* = 5.0, 9.9 Hz, 1H), 3.98 (t, *J* = 9.9 Hz, 1H), 3.86 (dd, *J* = 3.5, 10.1 Hz, 1H), 3.83–3.31 (m, 12H), 3.29 (s, 3H), 3.27 (s, 3H), 3.14 (sa, 1H, H-OH), 2.70 (d, *J* = 8.2 Hz, 1H), 2.43 (ddd, *J* = 4.4, 4.4, 13.2 Hz, 1H), 1.59 (q, *J* = 12.5 Hz), 1.34 (s, 3H), 1.34 (s, 3H), 1.05 (s, 9H), 0.97 (s, 9H); ^13^C NMR (CDCl_3_, 100 MHz) δ: 101.5, 99.8, 99.7, 98.5, 85.6, 81.2, 76.8, 74.0, 70.5, 70.4, 70.0, 69.4, 67.1, 66.4, 66.3, 65.9, 59.6, 59.1, 50.1, 48.1, 48.0, 32.0, 27.3 (3C), 26.9 (3C), 22.6 (2C), 17.7, 17.6; MS-API-ES: 865 [M + Na]^+^. HRMS (ESI^+^) calc for C_32_H_54_N_12_NaO_13_Si 865.35948, found 865.35922. **10b**: ^1^H NMR (CDCl_3_, 400 MHz) δ: 5.06 (d, *J* = 3.7 Hz, 2H), 4.51(d, *J* = 1.1 Hz), 4.20 (ddd, *J* = 5.0, 10.1, 10.1 Hz, 1H), 4.07 (dd, *J* = 4.2, 5.6 Hz, 1H), 4.04-3.96 (m, 2H), 3.88–3.72 (m, 4H), 3.69-3.29 (m, 9H), 3.28 (s, 3H), 3.26 (s, 3H), 2.62 (d, *J* = 8.3 Hz, 1H), 2.43 (ddd, *J* = 3.6, 3.6, 8.3 Hz, 1H), 2.36 (d, *J* = 2.3 Hz, 1H), 1.56 (q, *J* = 12.4 Hz, 1H), 1.34 (s, 6H), 1.31(s, 3H), 1.06 (s, 9H), 1.00 (s, 9H); ^13^C NMR (CDCl_3_, 100 MHz) δ: 100.9, 100.4, 99.6, 99.3, 87.3, 85.6, 81.9, 77.2, 73.4, 72.8, 70.6, 68.8, 68.7, 68.1, 67.1, 66.9, 66.7, 66.4, 59.9, 48.4, 48.1, 32.2, 27.4 (3C), 26.9 (3C), 22.7 (2C), 17.7, 17.6; MS-API-ES: 865 [M + Na]^+^. HRMS (ESI^+^) calc for C_32_H_54_N_12_NaO_13_Si 865.35948, found 865.35965.

#### 3.3.4. Synthesis of 5,2′,3″-*O*-tri-Benzyl-2′,3′-*O*-(2,3-butanedione-bis-dimethyl-acetal)-4″,6″-*O*-di-*tert*-butyl-silane-1,3,6′,3″-tetra-azido-kanamycin A (**11**)

To a stirred solution of **10a** (0.4 g, 0.38 mmol) and tetrabutylammonium iodide (51 mg, 0.14 mmol) in dry DMF (1.9 mL) at 0 °C was added NaH (60% oil suspension, 0.1 g, 2.85 mmol). The obtained mixture was stirred for 10 min at this temperature and then benzyl bromide (0.27 mL, 2.24 mmol) was added dropwise. The reaction mixture was stirred overnight at room temperature and then MeOH (1.5 mL) was added to quench. The solvent was eliminated under reduced pressure and the residue was diluted with brine, and extracted with CH_2_Cl_2_ (2 × 25 mL). The organic phases were dried over anhydrous Na_2_SO_4_, filtered and concentrated. Finally, the residue was purified by flash chromatography on silica gel using hexane/AcOEt (95:5) to give **11** (0.28 g, 65%) as a white solid. ^1^H NMR (CDCl_3_, 400 MHz) δ 7.45–7.00 (m, 15H, Ar), 5.46 (d, *J* = 4.1 Hz, 1H), 5.22 (d, *J* = 3.6 Hz, 1H), 5.04 (d, *J* = 11.2 Hz, 1H), 4.81 (d, *J* = 12.1 Hz, 1H), 4,76 (d, *J* = 11.3 Hz, 1H), 4.66 (d, *J* = 11.2 Hz, 1H), 4.63(d, *J* = 12.1 Hz, 1H), 4.36 (dd, *J* = 4.8, 2.4 Hz, 1H), 4.34 (d, *J* = 11.3 Hz, 1H), 4.14 (t, *J* = 10.1 Hz, 1H), 3.91 (dd, *J* = 10.0, 5.0 Hz, 1H), 3.82 (dd, *J* = 10.2, 5.3 Hz, 1H), 3.80 (t, *J* = 9.4 Hz, 1H), 3.64 (dd, *J* = 10.0, 2.5 Hz, 1H), 3.61–3.56 (m, 1H), 3.55–3.53 (m, 1H), 3.50–3.45 (m, 2H), 3.44 (m, 1H), 3.38 (dd, *J* = 6.7, 3.8 Hz, 1H), 3.32–3.26 (m, 2H), 3.24 (s, 3H), 3.25 (s, 3H), 3.20–3.17 (m, 2H), 3.16 (dd, *J* = 10.3, 3.5 Hz, 1H), 2.40–2.38 (m, 1H), 1.65–1.51 (m, 1H), 1.29 (s, 6H), 1.02 (s, 9H), 0.89 (s, 9H). ^13^C NMR (CDCl_3_, 100 MHz) δ: 138.4 (2C), 138.1, 129.2, 128.7 (2C), 128.4 (2C), 128.3 (2C), 128.1 (2C), 127.9 (2C), 127.7, 127.3, 126.4 (2C), 99.8, 99.6, 98.2, 97.4, 84.0, 81.4, 78.4, 77.1, 76.5, 74.4, 74.1, 73.9, 70.1, 69.4, 68.1, 67.6, 66.7, 64.7, 59.2, 50.6 (2C), 48.3, 48.2 (2C), 32.2, 31.7, 28.5, 27.6 (3C), 27.0 (3C), 22.8, 20.0. MS-API-ES: 1135 [M + Na]^+^. HRMS (ESI^+^) calc for C_53_H_72_N_12_NaO_13_Si 1035.50033, found 1035.50102.

#### 3.3.5. Synthesis of 5,2′,3″-*O*-tri-Benzyl-4″,6″-*O*-di-*tert*-butyl-silane-1,3,6′,3″-tetra-azido-kanamycin A (**12**)

To a solution of **11** (0.25 g, 0.22 mmol) in CH_2_Cl_2_ (2.3 mL) was added a mixture of TFA: H_2_O (1 mL, 10:1 *v*/*v*). The mixture was stirred at room temperature for 40 min at room temperature and then, a saturated aqueous solution of NaHCO_3_ was added and the mixture was extracted twice with AcOEt (25 mL). The combined organic phases were dried over anhydrous Na_2_SO_4_, filtered and the solvent was removed under reduced pressure. Finally, the residue was purified by flash chromatography on silica gel using hexane/AcOEt (7:3) to give **12** (0.27 g, 85%) as a white foam. ^1^H NMR (CDCl_3_, 400 MHz) δ 7.44–7.17 (m, 15H), 5.39 (d, *J* = 4.1 Hz, 1H), 5.34 (d, *J* = 3.4 Hz), 5.00 (d, *J* = 10.2 Hz, 1H), 4.93 (d, *J* = 11.2 Hz, 1H), 4.82 (d, *J* = 10.2 Hz, 1H), 4.66 (d, *J* = 11.8 Hz, 1H), 4.59 (d, *J* = 11.2 Hz, 1H), 4.20 (ddd, *J* = 9.9, 5.3, 2.4 Hz, 1H), 3.93–3.91 (m, 2H), 3.89 (m, 1H), 3.82 (dd, *J* = 9.8, 8.9 Hz, 1H), 3.69 (t, *J* = 9.4 Hz, 1H), 3.65 (m, 2H), 3.60 (m, 1H), 3.58 (ddd, *J* = 9.4, 5.6, 3.2 Hz, 1H), 3.55 (m, 1H), 3.49 (m, 1H), 3.42 (dd, *J* = 13.3 Hz, 1H), 3.32 (ddd, *J* = 5.7, 3.0, 1.3 Hz, 1H), 3.28 (m, 1H), 2.24 (dt, *J* = 12.5, 4.0 Hz, 1H), 1.36 (q, *J* = 12.5 Hz, 1H), 1.02 (s, 9H), 0.89 (s, 9H). ^13^C NMR (CDCl_3_, 100 MHz) δ: 138.6, 138.5, 138.3, 128.3 (2C), 128.2 (2C), 128.1 (2C), 128.0 (2C), 127.9 (2C), 127.8, 127.5, 127.2 (2C), 127.1, 97.8, 97.5, 83.9, 78.7, 78.6, 77.0, 76.8, 74.5, 74.1, 73.8, 73.2, 72.2, 70.9, 67.6, 66.6, 64.9, 59.8, 59.6, 51.7, 46.8, 31.9, 26.7 (3C), 26.3 (3C), 22.3, 19.5. MS-API-ES: = 999 [M + H]^+^. HRMS (ESI^+^) calc for C_47_H_63_N_12_O_11_Si 999.45030, found 999.45168.

#### 3.3.6. Synthesis of 1,3-di-Azido-5-*O*-benzyl-[(3-deoxy-3-azido-2-*O*-Benzyl-4,6-*O*-di-*tert*-butyl-silane)-α-d-glucopyranosyl]-2-deoxy-streptamine (**13**)

To a well stirred solution of **12** (0.27 g, 0.27 mmol) in MeOH (8.8 mL) at 0 °C was added NaIO_4_ (0.1 g, 0. 52 mmol). The resulting suspension was stirred at this temperature for 24 h and then the mixture was filtered through Celite^®^, which was washed with AcOEt. The mixture was concentrated and the residue was dissolved in THF (6.6 mL) and treated with TEA (110 ∝L, 0.79 mmol). The reaction was stirred under Ar at room temperature overnight and then the solvent was eliminated under vacuum. The residue was purified by flash chromatography on silica gel using hexane/AcOEt (9:1) to give **13** (0.17 g, 88%) as a white solid. ^1^H NMR (CDCl_3_, 500 MHz) δ: 7.44–7.28 (10H, m), 5.43 (1H, d, *J* = 3.8 Hz), 5.0 (1H, d, *J* = 11.8 Hz), 4.81 (1H, d, *J* = 12.0 Hz), 4.68 (1H, d, *J* = 11.1 Hz), 4.10 (1H, dd, *J* = 5.1, 10.0 Hz), 4.03 (1H, ddd, *J* = 5.1, 9.9, 9.9), 3.88 (1H, dd, *J* = 9.5, 10.1 Hz), 3.80 (1H, dd, *J* = 10.1, 10.1 Hz), 3.75 (dd, *J* = 9.5, 9.5 Hz, 1H), 3.55–3.30 (5H, m), 3.30 (dd, *J* = 3.8, 10.3 Hz, 1H), 2.22 (m, 1H), 2.21 (d, *J* = 2.4 Hz, 1H), 1.41 (m, 1H), 1.06 (s, 9H), 0.94 (s, 9H). ^13^C NMR (CDCl_3_, 125 MHz) δ: 138.1, 137.7, 128.9 (2C), 128.5 (2C), 128.2, 127.94, 127.92 (2C), 127.6 (2C), 96.9, 83.2, 79.3, 77.0, 76.9, 76.4, 75,3, 73.4, 67.1, 66.6, 64.8, 60.4, 59.7, 31.9, 27.6 (3C), 26.9 (3C), 22.7, 19.8. MS-API-ES: 744.3 [M + Na]^+^. HRMS (ESI^+^) calc for C_34_H_47_N_9_NaO_7_Si 744.32599, found 744.32621.

#### 3.3.7. Synthesis of 6-*O*-[(3-Deoxy-3-amino)-α-d-glucopyranosyl]-2-deoxy-streptamine (**7**)

To a solution of **13** (0.17 g, 0.23 mmol) in dry THF (5 mL) was added dropwise TBAF (1M, THF) (1.15 mL, 1.15 mmol). The reaction was stirred under Ar at room temperature for 24 h and then the mixture was concentrated under reduced pressure. The residue was dissolved in THF and an aqueous solution of 0.1 M NaOH (0.44 mL) and a 1.0M solution of PMe_3_ in THF (1.25 mL) were sequentially added. The solution was stirred at 55 °C overnight and then MeOH was added (3 mL). The mixture was concentrated under reduced pressure and the residue was filtered through a pad of silica gel using NH_4_OH/BuOH/EtOH/toluene (4:3.5:2:1) as eluent. The obtained compound was dissolved in a mixture of H_2_O/TFA (4.4 mL, 1:1 *v*/*v*) and a catalytic amount of Pd(OH)_2_/C (20%) was added. The reaction mixture was stirred under H_2_ atmosphere (balloon), and then filtered through Celite^®^ and the filtrate was concentrated under reduced pressure. Finally, the residue was purified by flash chromatography on silica gel using NH_4_OH/BuOH/EtOH/CH_2_Cl_2_ (4:3:2.5:1) followed by an ion exchange chromatography on Amberlite^®^ CG-50 using NH_4_OH (0.5M) to give **7** (40 mg, 47%) as a white foam. ^1^H NMR (D_2_O, 500 MHz) δ: 4.91 (d, *J* = 3.9 Hz, 1H), 3.80 (dt, *J* = 10.2, 3.4 Hz, 1H), 3.69–3.60 (m, 2H), 3.41 (dd, *J* = 10.5, 3.9 Hz, 1H), 3.34–3.19 (m, 2H), 3.21–3.12 (m, 2H), 2.92 (t, *J* = 10.1 Hz, 1H), 2.84 (ddd, *J* = 12.1, 9.7, 4.1 Hz, 1H), 2.74 (ddd, *J* = 12.2, 9.9, 4.2 Hz, 1H), 1.91 (dt, *J* = 12.9, 4.3 Hz, 1H), 1.19 (q, *J* = 12.4 Hz, 1H). ^13^C NMR (D_2_O, 125 MHz) δ: 100.0, 86.9, 76.3, 74.0, 71.9, 71.3, 68.7, 60.0, 54.2, 50.5, 50.3, 33.8. MS-API-ES: 324 [M + H]^+^. HRMS (ESI^+^) calc for C_12_H_26_N_3_O_7_ 324.17653, found 324.17635.

### 3.4. MIC Determination

The selected bacterial strains were grown in Mueller–Hinton broth (1 mL) until an optical density at 600 nm (OD_600_) of 0.5 units. At this time, kanamycin A (**1**), neamine (**4**) and *pseudo*-disaccharide **7**, as free-bases, were added from stock solutions (0.5–400 ∝g/mL) prepared at different concentrations. These cultures were incubated at 37 °C for 24 h, after which the OD_600_ of each sample was recorded. Herein, we considered the MIC as the lowest concentration of aminoglycoside which produced an inhibition of the bacterial growth greater than 90%. For resistant strains with overexpressed AAC-(6′)-Ib, ANT(4′) or APH-(3′) enzymes, *E. coli* (BL21) with the corresponding plasmids (pET-AAC(6′)-Ib, pET-ANT-(4′), and pET-APH-(3′)) were induced with IPTG before adding the aminoglycosides.

### 3.5. Enzymatic Activity

#### 3.5.1. Enzymatic Activity of ANT-(4′) from *S. aureus* by HPLC Assay

To a solution of a total volume of 1 mL containing variable concentrations of kanamycin A (**1**), neamine (**4**) or compound **7** (0.03–3 mM) in sodium phosphate buffer (20 mM, pH 7.5) containing MgCl_2_ (5 mM) and ATP (3 mM) was added the ANT-(4′) enzyme (0.5 U). The formation of the final products (AMP-kanamycin, AMP-neamine or AMP-compound **7**) was monitored by HPLC (Vydac 30/I, TFA PH = 3, ø = 1 mL·min^−1^, λ = 260 nm), and confirmed by mass spectrometry of the final reaction mixtures. In the spectrum we observed the presence of the corresponding adenylated products. AMP-**1**: 814.7 [M + H]^+^; AMP-**4**: 652.7 [M + H]^+^; AMP-**7**: 653.7 [M + H]^+^.

#### 3.5.2. Enzymatic Activity of APH-(3′) from *E. coli* by ELISA

A solution of a total volume of 1 mL containing variable concentrations of kanamycin A (**1**), neamine (**4**) or compound **7** (5 μM to 1.25 mM) in Hepes (50 mM, pH 7.7) containing KCl (40 mM), MgCl_2_ (10 mM), NADH (247 μM), phosphoenolpyruvate (2.5 mM), ATP (1 mM) and pyruvate kinase/lactate dehydrogenase enzyme (5 U) was incubated at 37 °C for 5 min. Subsequently, the APH-(3′)-III (10 U) was added. The phosphorylation of kanamycin A (**1**), neamine (**4**) and compound **7** was monitored by a spectrophotometric experiment (λ = 340 nm, ε = 6220 M^−1^·cm^−1^). Blank assays were prepared in identical conditions but in the absence of the aminoglycosides and their spectrophometric readings were subtracted from the reaction readings.

#### 3.5.3. Enzymatic Activity of AAC-(6′) from *S. aureus* by ELISA

To a solution of a total volume of 500 ∝L containing DTP (2 mM), acetyl-CoA (40 μM), EDTA (1 mM), and the enzyme (10 μL at 5 mg mL^−1^) in Tris buffer (pH = 7.6, 50 mM) kanamycin (**1**), neamine (**4**) or derivative **7** was added (0.1 mM–1 mM). Subsequently, the reaction mixtures were stirred at 37 °C for 30 min. During the reaction, spectrophotometric readings were taken every 30 s (λ = 324 nm).

### 3.6. Thermal Shift Assay

Thermal melting shift experiments were conducted on an iQ5 Real Time Detection System (Bio-Rad, Foster City, CA, USA) using the fluorescent dye SYPRO Orange [29]. In a typical experiment, a solution of SYPRO Orange (2.8 mM) and protein (ANT-(4′), APH-(3′)-III and AAC-(6′)-Ib)) (4.4 ∝M) in Tris buffer (10 mM, pH 8.0, 0.2 mM NaCl, 90 ∝L) was mixed with a solution of the corresponding aminoglycoside (**1**, **4**, **7**) (10 ∝M, 100 ∝L). The final mixtures were heated from 25 °C to 70 °C with a heating rate of 0.5 °C min^−1^, measuring the fluorescence after each heating step with an Ex/Em 490/530 nm.

## Figures and Tables

**Table 1:** MIC values of kanamycin A (**1**), neamine (**4**) and compound **7** against non-resistant and resistant strains.

Entry	Strain	MIC (µg mL^−1^) Compounds		
		1	4	7
**1**	*E. coli*/ATCC 2592	4	100	50
**2**	*E. coli* BL21	6	100	50
**3**	*B. cereus*/ATCC 117781	1.5	100	50
**4**	*S. epidermis*/ATCC 1228	>100	>50	50
**5**	*E. coli* (APH-3″IIa)	>200	>200	100
**6**	*E. coli* (ANT-4′)	>200	>200	100
**7**	*E. coli* (AAC-6′)	>200	>200	100

**Table 2:** Tm values of resistant enzymes with and without kanamycin A (**1**), neamine (**4**) and compound **7**.

Enzyme	Blank	1	4	7
ANT-(4′)	58 ± 0.1	61.5 ± 0.1	61 ± 0.1	59 ± 0.1
AAC-(6′)	44 ± 0.09	49 ± 0.1	48 ± 0.2	49 ± 0.1
APH-(3′)	49 ± 0.09	62 ± 0.1	56 ± 0.2	56 ± 0.1

## Supplementary Materials

The following are available online at https://www.mdpi.com/2079-6382/8/3/109/s1. Figure S1: 1D- and 2D-NMR spectra of pseudo-disaccharide (**7**).
