# Supplementary material for: Synthesis of Ring II/III Fragment of Kanamycin: A New Minimum Structural Motif for Aminoglycoside Recognition
Source: Antibiotics (Basel). 2019 Aug 2;8(3):109. doi: 10.3390/antibiotics8030109 (PMC6783941; doi:10.3390/antibiotics8030109)
Supplement: Supplementary file 1 [file antibiotics-08-00109-s001.pdf]

Pseudo-desprotegido-1H
STANDARD PROTON PARAMETERS

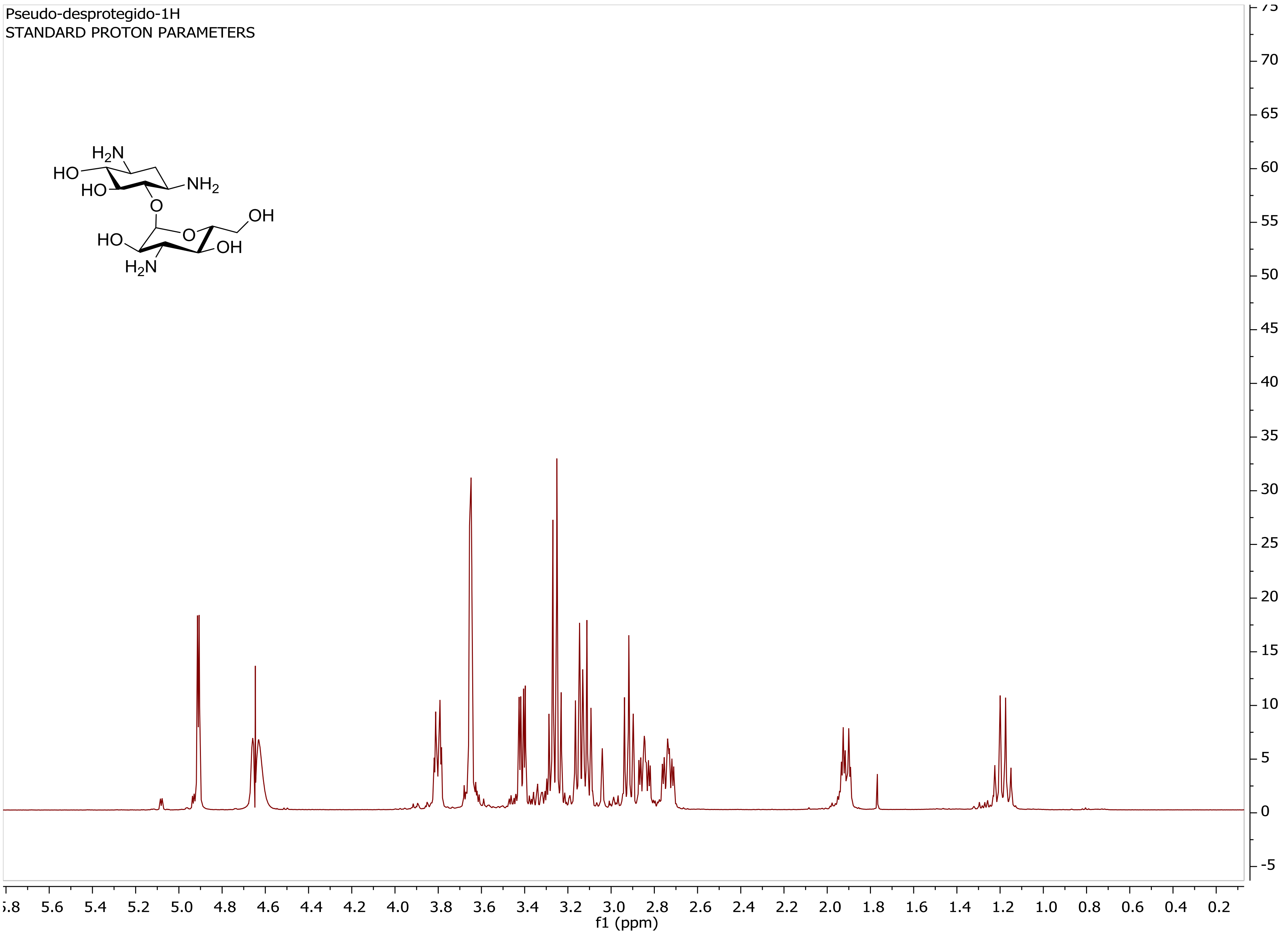

Pseudo-desprotegido-13C
STANDARD PROTON PARAMETERS

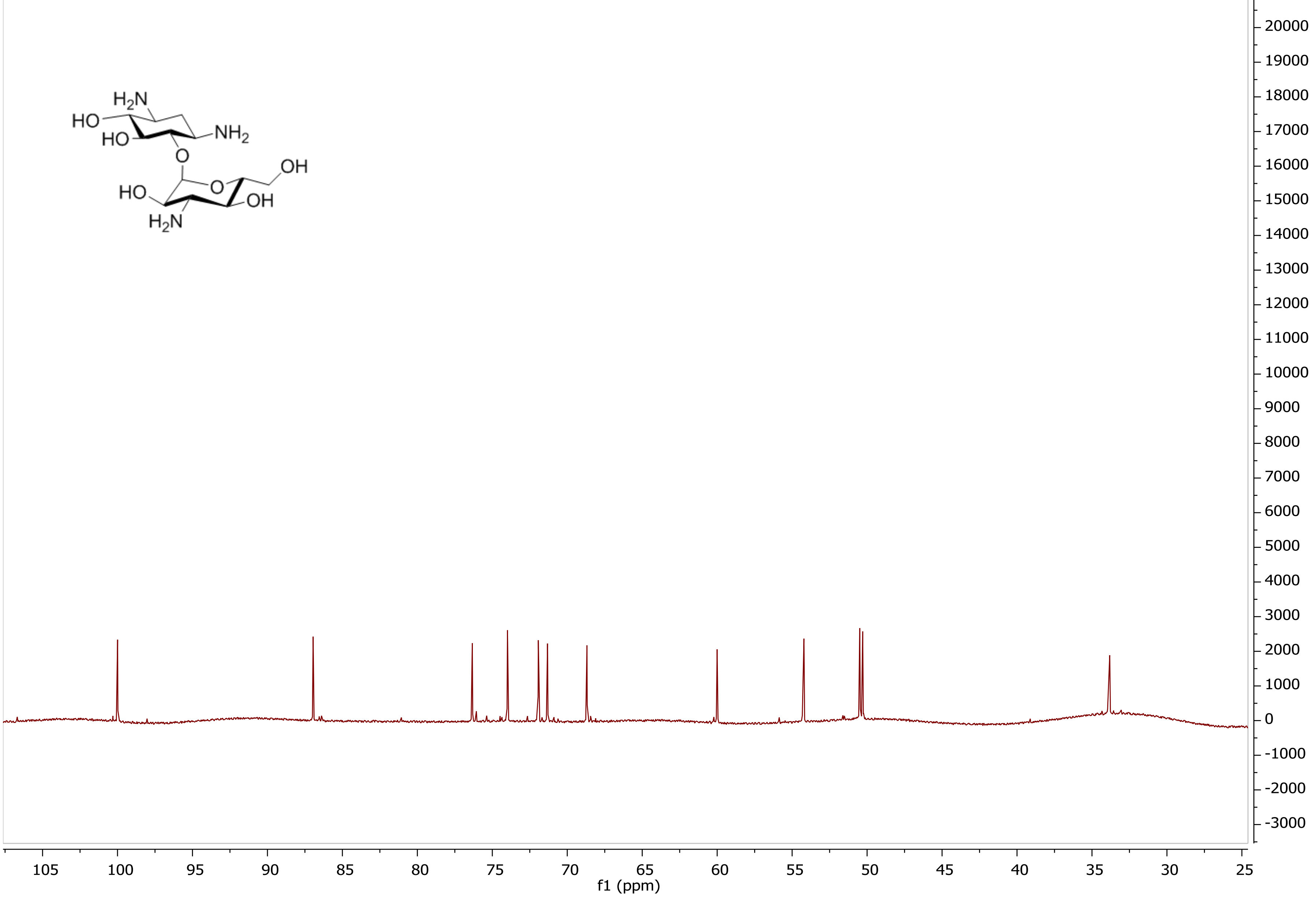

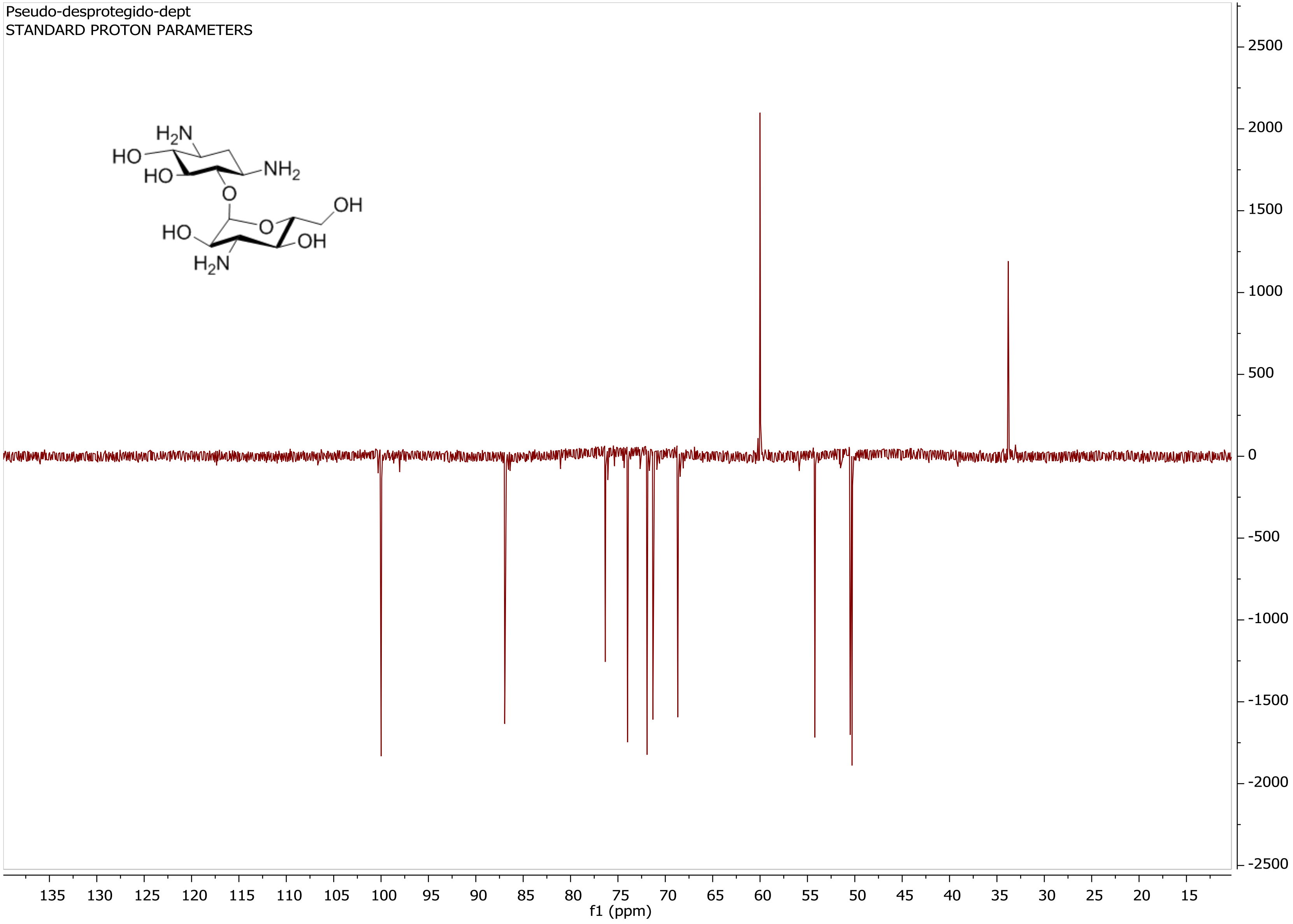

Pseudo-desprotegido-dept
STANDARD PROTON PARAMETERS
H2N
HO
HO
NH2
O
OH
O
HO
OH
H2N
2500
2000
1500
1000
500
0
-500
-1000
-1500
-2000
-2500
135 130 125 120 115 110 105 100 95 90 85 80 75 70 65 60 55 50 45 40 35 30 25 20 15
f1 (ppm)

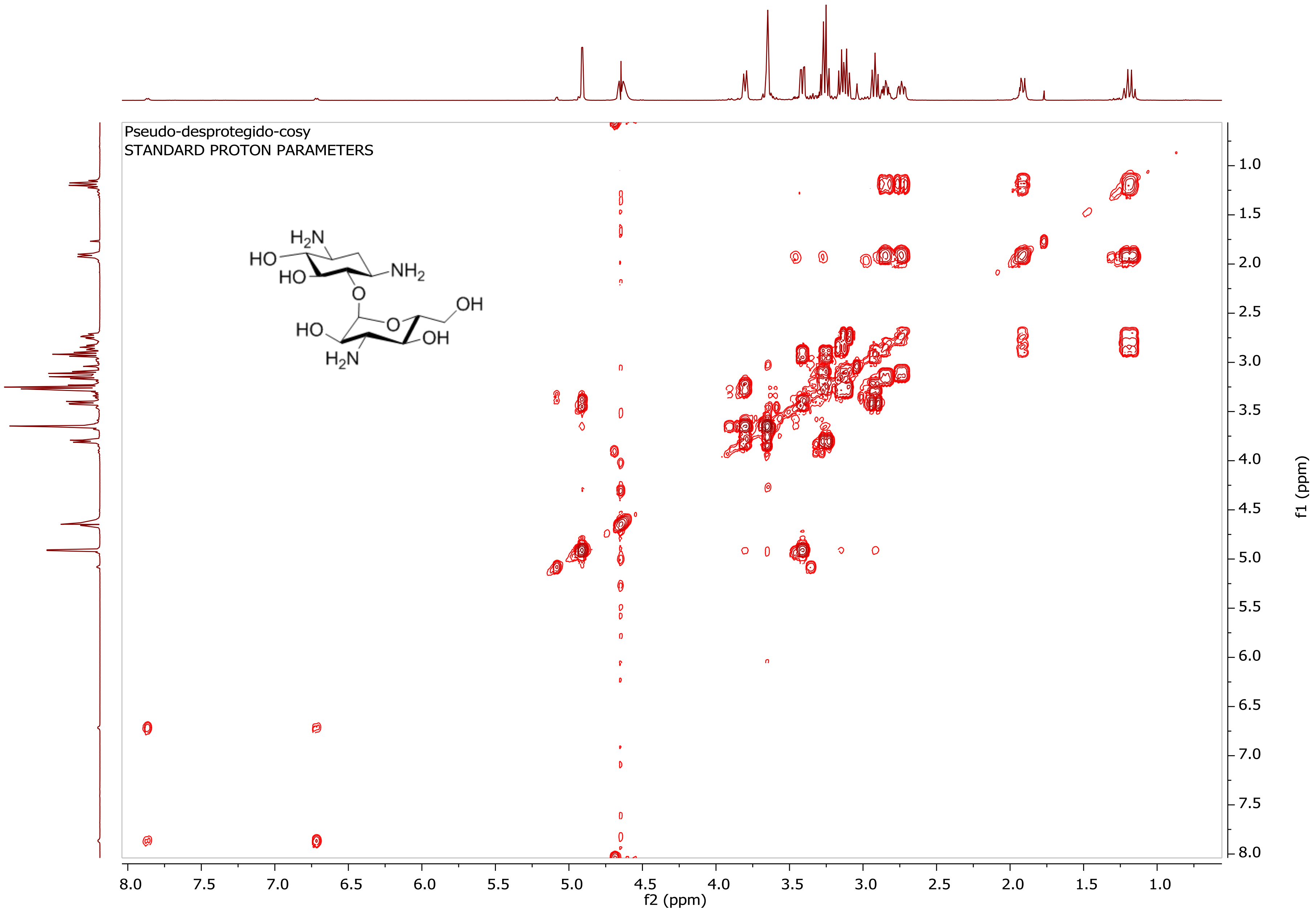

Pseudo-desprotegido-cosy
STANDARD PROTON PARAMETERS
H2N
HO
HO
NH2
O
OH
O
HO
OH
H2N
f2 (ppm)
f1 (ppm)
8.0 7.5 7.0 6.5 6.0 5.5 5.0 4.5 4.0 3.5 3.0 2.5 2.0 1.5 1.0

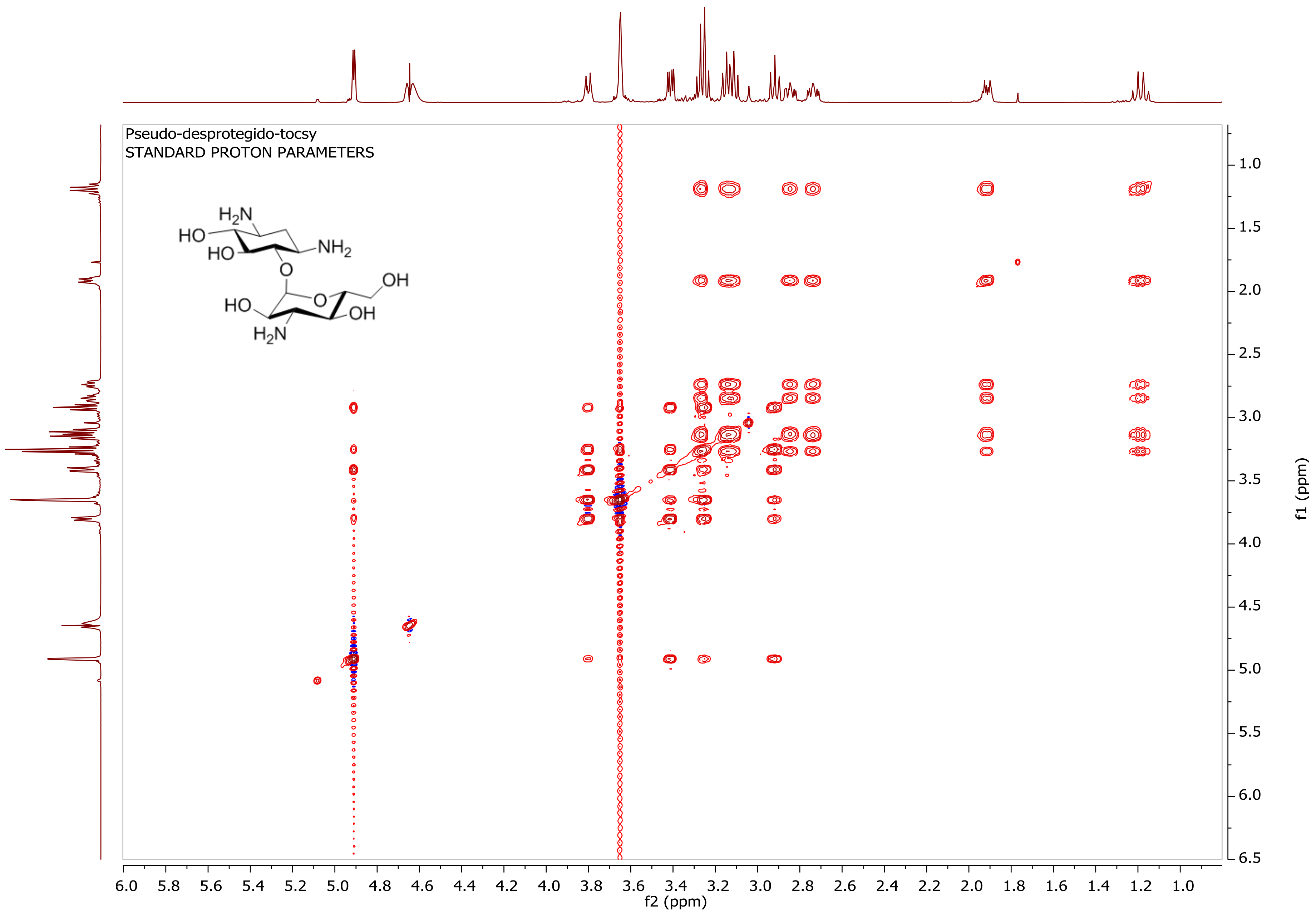

Pseudo-desprotegido-tocsy
STANDARD PROTON PARAMETERS
H2N
HO
HO
NH2
O
OH
O
HO
OH
H2N
f2 (ppm)
f1 (ppm)

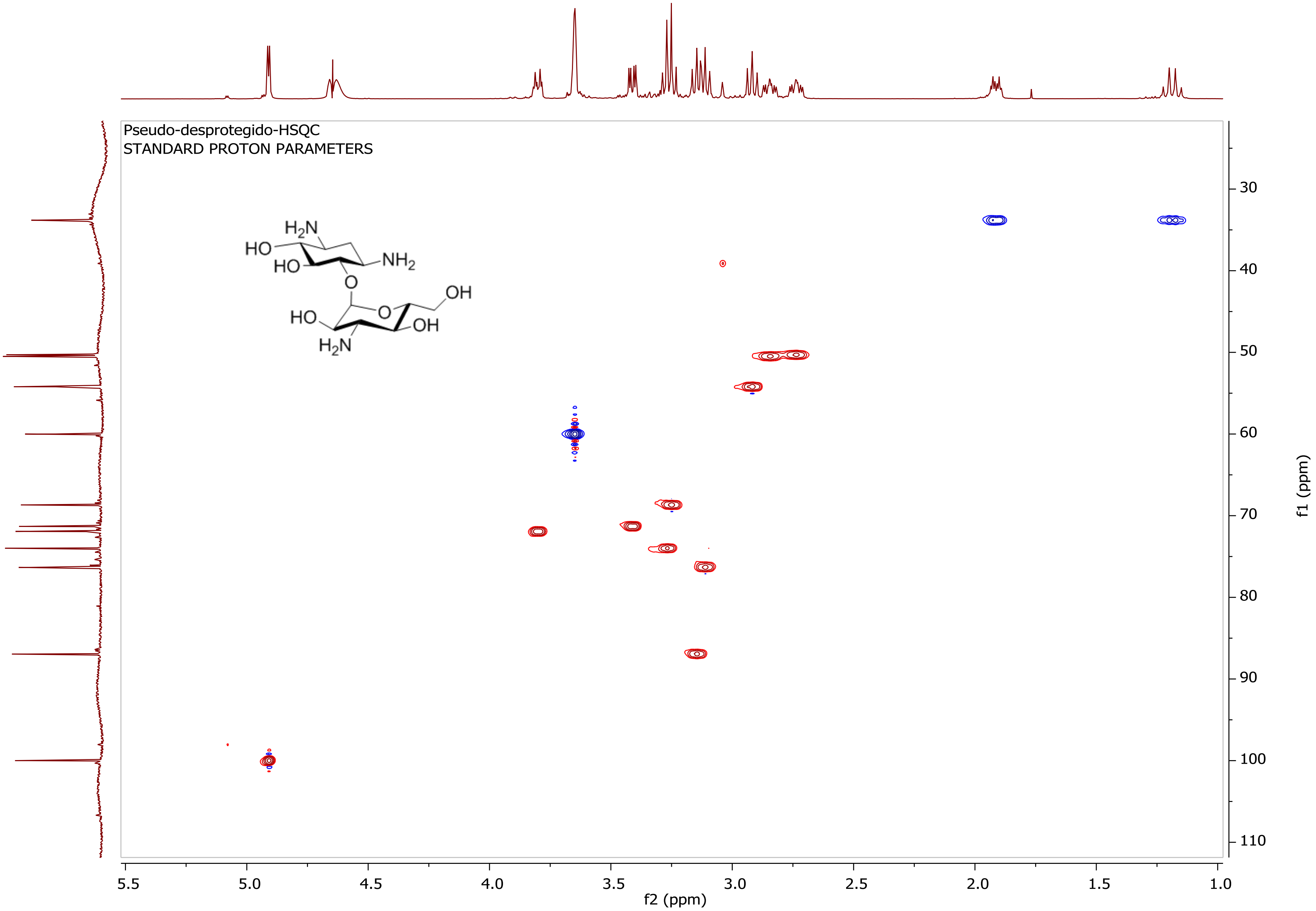

Pseudo-desprotegido-HSQC
STANDARD PROTON PARAMETERS
H2N
HO
HO
NH2
O
OH
HO
O
OH
H2N
f2 (ppm)
f1 (ppm)
5.5
5.0
4.5
4.0
3.5
3.0
2.5
2.0
1.5
1.0
30
40
50
60
70
80
90
100
110
